# Secondary Abdominal Cocoon Syndrome Due To Chronic Beta-Blocker Use

**DOI:** 10.7759/cureus.10509

**Published:** 2020-09-17

**Authors:** Jennifer C Asotibe, Pejman Zargar, Ikechukwu Achebe, Benjamin Mba, Vikram Kotwal

**Affiliations:** 1 Medicine, John H. Stroger, Jr. Hospital of Cook County, Chicago, USA; 2 Gastroenterology, John H. Stroger, Jr. Hospital of Cook County, Chicago, USA; 3 Internal Medicine, John H. Stroger, Jr. Hospital of Cook County, Chicago, USA

**Keywords:** beta blocker side effects, abdominal cocoon, sclerosing encapsulating peritonitis, bowel obstruction, propranolol

## Abstract

Sclerosing encapsulating peritonitis (SEP), which is interchangeably used with the term ‘’abdominal cocoon syndrome’’, is a rare condition characterized by a thick fibrous membrane encasing portions of the intestinal wall leading to recurrent bowel obstructions. To date, literature describing the association between this condition and chronic beta-blocker therapy is scarce. This report adds by detailing a rare presentation of SEP and highlights an understudied yet important association of SEP with chronic beta-blocker therapy.

## Introduction

Sclerosing encapsulating peritonitis (SEP) is characterized by a thick fibrous membrane encasing portions of the intestinal wall and presenting with recurrent bowel obstructions [1]. While this condition is often asymptomatic, patients may present with moderate-to-severe abdominal pain due to partial or complete bowel obstruction.

These patients often undergo multiple corrective surgeries, increasing patients’ overall discomfort and decreasing their quality of life. SEP requires early diagnosis for the optimal management and reduction of morbidity. This report adds by highlighting an association between the development of this condition and chronic beta-blocker (BB) use.

## Case presentation

A 64-year-old male with a past medical history of Child B compensated liver cirrhosis complicated by non-bleeding esophageal varices, portal vein thrombosis, diverticulosis, and history of episodes of small bowel obstruction presented with chronic diffuse abdominal pain for three months. Review of systems was negative for nausea, vomiting, diarrhea, constipation, fever, or chills. The patient was passing flatus but denied any bowel movements for four days.

On physical examination, bowel sounds were decreased, and the patient’s abdomen was diffusely tender to palpation. Heart sounds were normal, and lungs were clear to auscultation.

Review of medication history revealed that the patient had been taking propranolol for primary prevention of variceal bleed for three years since being diagnosed with cirrhosis.

Pertinent laboratory values were as follows: sodium = 133 mEq/L (low [L]), potassium = 4.4 mEq/L (normal [nl]), chloride = 105 mEq/L (nl), BUN (blood urea nitrogen) = 16 mg/dL (nl), creatinine = 0.9 mg/dL (nl), white blood cell = 3.9 k/uL (L), hemoglobin = 9.0 g/dL (L), platelet = 204 k/uL (nl), lactate = 2.8 mmol/L (high [H]), AST (aspartate aminotransferase) = 53 U/L (H), ALT (alanine aminotransferase) = 16 U/L (H), GGT (gamma-glutamyl transpeptidase) = 223 U/L (H), direct bilirubin = 0.3 mg/dL (H), total bilirubin = 1.1 mg/dL (nl), alkaline phosphatase = 160 U/L (H), total protein = 6.0 g/dL (L), albumin = 3.2g/dL (L), and bicarbonate = 22 mEq/L (L). 

Initial workup included an abdominal CT scan, which showed mobile loops of bowel with air-fluid levels concerning for ileus versus small bowel obstruction. However, given that the patient was passing gas, concern for complete bowel obstruction was low, and conservative medical management was recommended by the consulted surgical team. 

Review of the previous medical records showed this patient to have a history of recurrent admissions for unremitting, diffuse abdominal pain. During a surgery to relieve his bowel obstruction approximately two years prior, a thick fibrous tissue encapsulating the ileum, jejunum, gallbladder, stomach, transverse colon, and omentum was noticed. Additionally, a separate fibrous capsule was found that encapsulated the aforementioned structures and fibrous sheath. The distal ileum was found to be completely decompressed, consistent with a diagnosis of small bowel obstruction. To relieve the obstruction, the thick fibrous tissue encasing the ileum and jejunum had to be excised. Some of the fibrous casing had to be left intact to preserve the integrity of bowel tissues.

**Figure 1:**
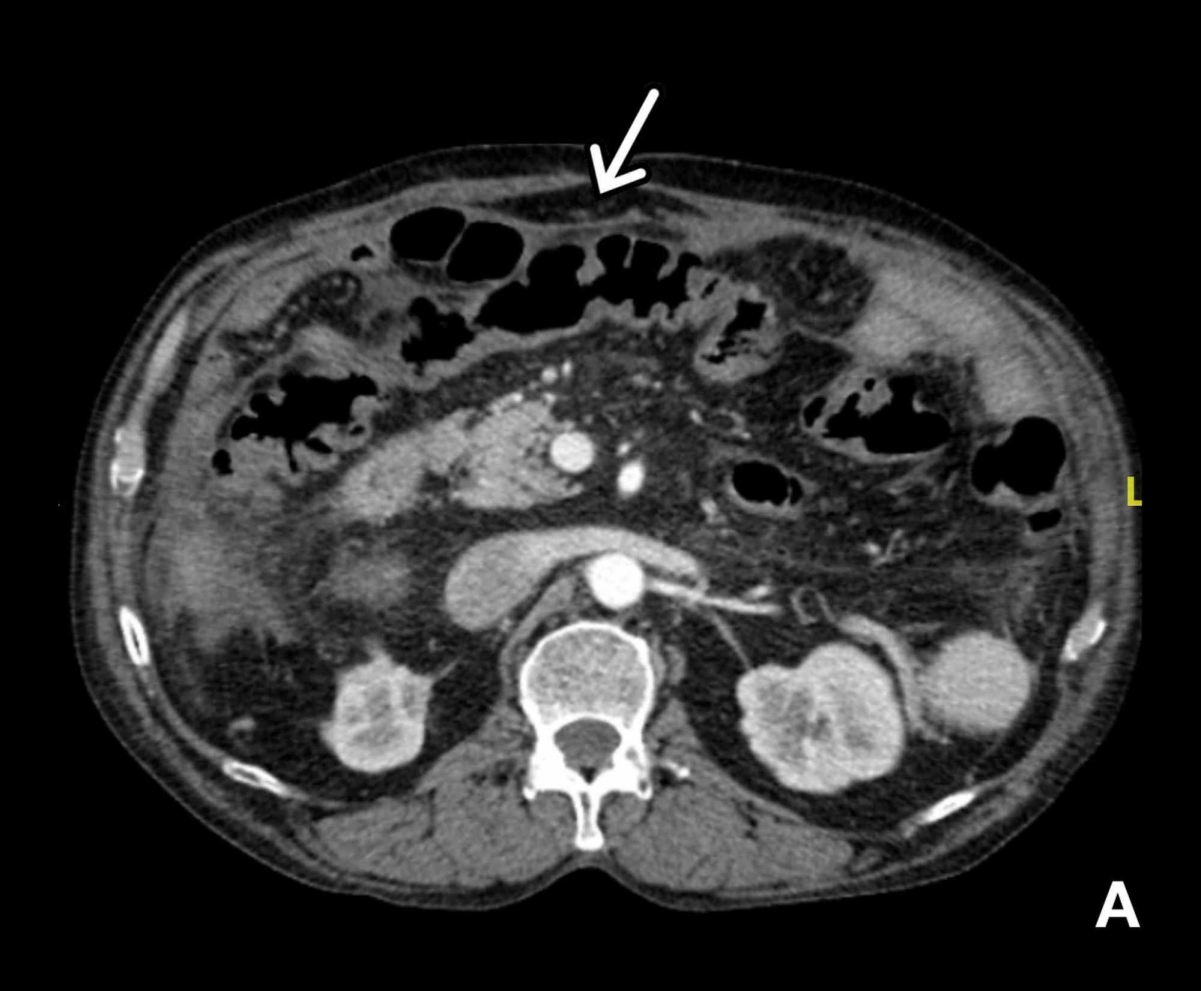
Axial view of CT of the abdomen, with arrow pointing to fibrous tissue encasing large bowel

**Figure 2:**
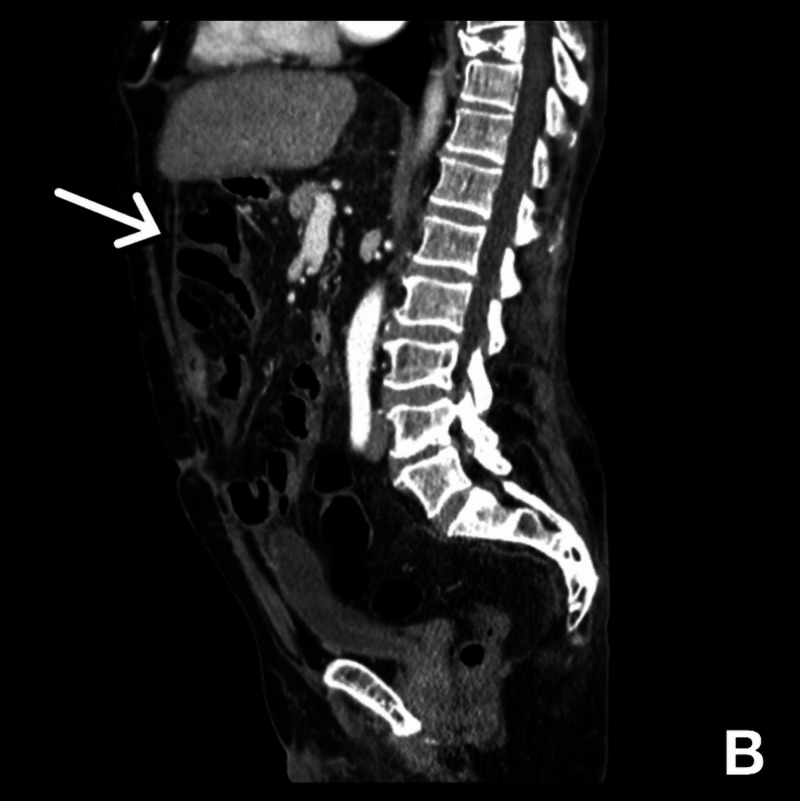
Sagittal view of CT of the abdomen, with arrow pointing to fibrous tissue encasing the stomach, small intestine, gall bladder, omentum, and large intestine

CT images from two years prior are shown in Figures 1, 2.

During this admission, however, the patient’s bowel obstruction was managed conservatively. By day 5 of his hospital admission, imaging showed resolution of the obstruction.

## Discussion

SEP is a rare condition characterized by thick fibrous membranes covering most or all of the small and large bowels [1]. SEP can be divided into two subtypes: idiopathic and secondary. While the pathogenesis of Idiopathic SEP is poorly understood, studies suggest that the fibrosis occurs secondary to unregulated fibroblasts activity and cytokine release. Additional theories postulate that because SEP is often accompanied by embryologic abnormalities, including omental hypoplasia, it may occur as a consequence of a developmental disorder [2-10]. While retrograde menstruation with a superimposed viral infection has been thought to cause this condition, the fact that a majority of patients with SEP are men, preadolescent, and postmenopausal women makes this less likely [2]. Idiopathic SEP was first described as “abdominal cocoon syndrome” by Foo et al. in 1968 [10]. It is classified into three categories depending on the extent of abdominal encasement. Encasement of only part of the intestine is seen in type I. In type II, the entire intestine is encased. In type III, both intestines and surrounding organs are encased. Idiopathic SEP is most common in tropical regions like India, South Africa, Nigeria, China, Malaysia, and Kenya. 

Secondary SEP is a term used to describe a secondary cause as a known precipitant. The most common cause of secondary SEP is peritoneal dialysis [3]. Other common causes include tuberculosis, chronic BB use, autoimmunity, ventriculoperitoneal shunts, protein S deficiency, and methotrexate use [2,3]. Secondary SEP is more common than the idiopathic variant and accounts for 70% of SEP cases. 

SEP or “abdominal cocoon syndrome” is often misidentified as a variant of abdominal encapsulation syndrome called congenital peritoneal encapsulation [4]. These conditions, however, are different. In abdominal cocoon syndrome, bowel loops are usually adherent to each other, and laparotomy usually reveals thick fibrous tissue encapsulating the entire bowel. In congenital peritoneal encapsulation, bowel loops typically do not adhere to each other. On laparotomy, these patients usually have a thin membranous tissue similar in texture to the peritoneum. 

Congenital peritoneal encapsulation was first described by Cleland in 1868 as a congenital anomaly that occurs secondary to abnormal return of abdominal wall contents during the 12th week of gestation [5]. The yolk sac coat returns with the abdominal wall contents instead of remaining on the umbilical pedicle leading to the membranous coat encasing the intestine. 

Clinical features of SEP range from recurrent episodes of abdominal pain, nausea, anorexia, to partial/complete bowel obstruction. Patients with SEP also have been described as having palpable, non-tender abdominal wall masses [6]. Some patients, however, are asymptomatic from SEP and are diagnosed incidentally during laparotomy for other conditions. Most commonly, this disease is found in patients undergoing peritoneal dialysis who present with new-onset bowel obstruction. The combination of prolonged exposure to dialysate fluid, acetate buffered dialysate fluid, higher concentration of glucose in dialysate fluid, and recurrent spontaneous bacterial peritonitis predisposes these patients to develop SEP [2]. A prospective multicenter study conducted in Japan by Kawanishi et al. showed an incidence of 2.5% after four years of undergoing peritoneal dialysis [7-9]. 

The relationship between BBs and SEP was first described in 1974 by Brown et al. after three female patients with SEP had no other significant risk factors except for the use of the BB proctalol [7]. Since then, increased SEP risk with other BBs including atenolol, timolol, and propanol is been reported. BBs are thought to enhance collagen production in these patients. Additionally, some patients on BB therapy develop adverse reactions leading to fibrosis of the peritoneum [1]. The patient reported in this case had no other risk factors for the development of SEP, and thus we were inclined to believe that his chronic BB use may have led to this condition. 

Diagnosis of SEP is usually based on patient presentation, comorbid risk factors, and imaging studies. X-rays, barium swallow, ultrasound, CT scan, and MRI are imaging modalities that assist in the diagnosis of SEP. Barium swallow typically will show a ‘’cauliflower appearance’’ of ileal loops clumped together. CT of the abdomen can provide more in-depth imaging of the small bowel loops encased by a thick membrane [8,9]. 

SEP can be managed conservatively, medically, or surgically. Conservative management involves nasogastric tube decompression, IV hydration, and bowel rest. Medications including tamoxifen, colchicine, azathioprine, mycophenolate, angiotensin II inhibitors, and steroids have been shown in the literature to aid treatment [2,6]. Patients who present with severe bowel obstruction or those who fail medical management typically require surgical intervention, which involves excision of the encapsulating membrane, enterolysis, and adhesiolysis [2,6]. While mortality rates can reach as high as 50% in the weeks following surgery, complete resolution of symptoms with little or no recurrence is common.

The rarity of SEP makes diagnosis difficult. Even after undergoing partial excision of the fibrous membranes causing his bowel obstruction two years prior, our patient continued to have symptoms and progression of his disease, likely as a consequence of continued BB use. Other possible explanations for his recurrent bowel obstruction include incomplete excision of the fibrous membrane or the formation of adhesions secondary to his intraabdominal surgery. Nevertheless, this case highlights an important association between the development of SEP and chronic BB use. If identified early, risk factors leading to disease progression can be mitigated early, improving patient well-being, morbidity, and mortality.

## Conclusions

SEP is a rare condition that causes recurrent small bowel obstruction and severe abdominal pain. It is important to diagnose this condition early and recognize chronic BB therapy as a potential cause of peritoneal fibrous tissue development. More reports describing this disease are needed to expand on the available knowledge surrounding SEP and aid in its early diagnosis and treatment.
